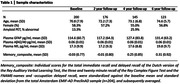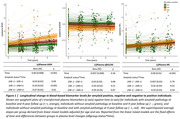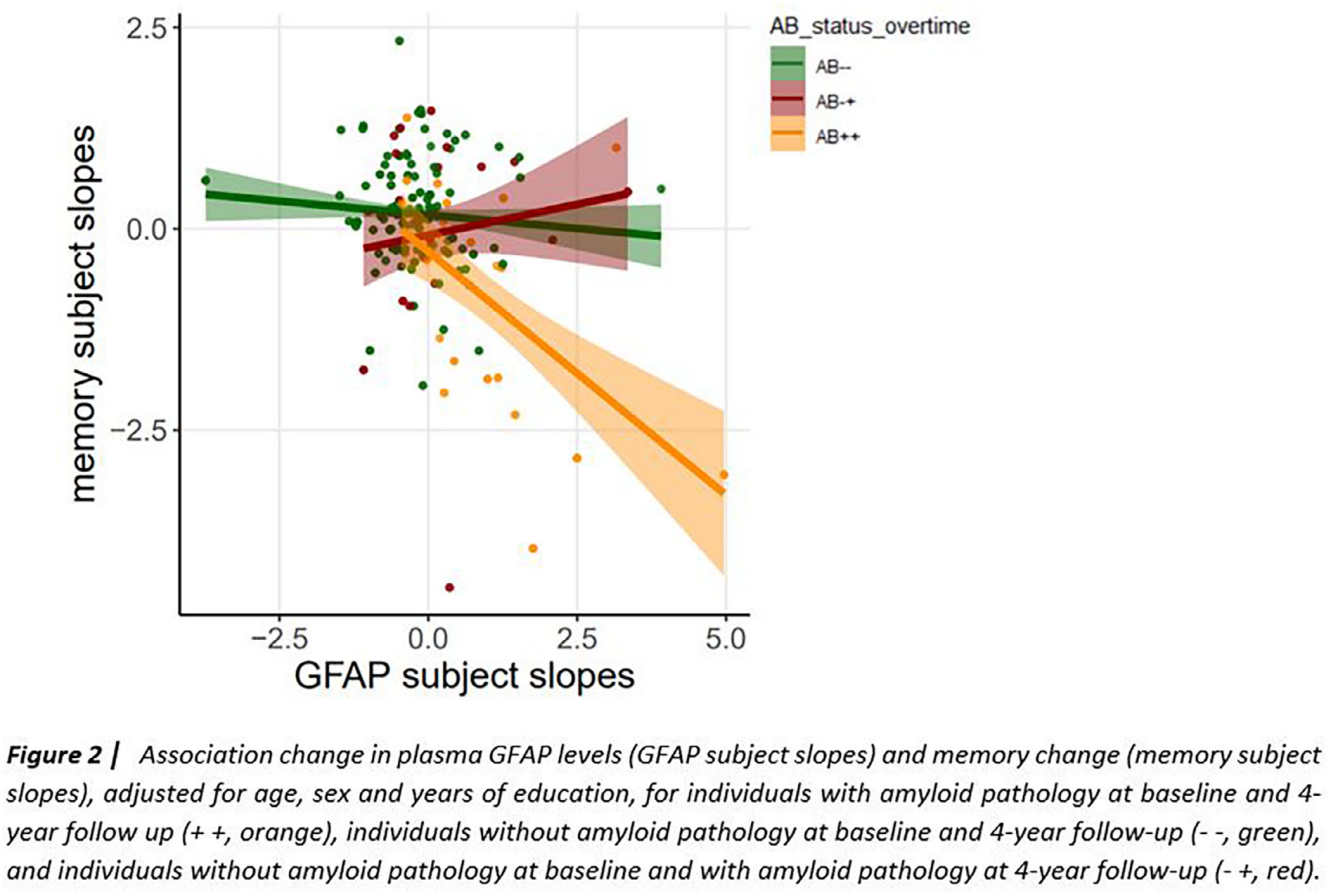# Changes in plasma biomarker levels are predictive of amyloid pathology and memory decline in cognitively unimpaired individuals

**DOI:** 10.1002/alz.090269

**Published:** 2025-01-09

**Authors:** Anouk den Braber, Rebecca Z. Rousset, Inge M.W. Verberk, Lynn Boonkamp, Jori Tomassen, Sophie M. van der Landen, Lyduine E. Collij, Frederik Barkhof, Elsmarieke van de Giessen, David H Wilson, Mike Miller, Yolande A.L. Pijnenburg, Wiesje M. van der Flier, Eco J.C. de Geus, Pieter Jelle Visser, Charlotte Teunissen

**Affiliations:** ^1^ Department of Biological Psychology, Vrije Universiteit Amsterdam, Amsterdam Netherlands; ^2^ Amsterdam Neuroscience, Neurodegeneration, Amsterdam Netherlands; ^3^ Alzheimer Center Amsterdam, Neurology, Vrije Universiteit Amsterdam, Amsterdam UMC location VUmc, Amsterdam Netherlands; ^4^ Neurochemistry Laboratory, Department of Laboratory medicine, Vrije Universiteit Amsterdam, Amsterdam UMC location VUmc, Amsterdam Netherlands; ^5^ Neurochemistry Laboratory, Department of Clinical Chemistry, Amsterdam Neuroscience, Vrije Universiteit Amsterdam, Amsterdam UMC, Amsterdam Netherlands; ^6^ Department of Radiology & Nuclear Medicine, Vrije Universiteit Amsterdam, Amsterdam UMC location VUmc, Amsterdam Netherlands; ^7^ Amsterdam Neuroscience, Brain Imaging, Amsterdam Netherlands; ^8^ Lund University, Lund Sweden; ^9^ UCL Institute of Neurology, London UK; ^10^ Quanterix, billerica, MA USA; ^11^ Quanterix Corporation, Billerica, MA USA; ^12^ Alzheimer Center Limburg, School for Mental Health and Neuroscience, Maastricht University, Maastricht Netherlands; ^13^ Department of Neurobiology, Care Sciences and Society, Division of Neurogeriatrics, Karolinska Institutet, Stockholm, Stockholm Sweden; ^14^ Amsterdam UMC, Amsterdam Netherlands

## Abstract

**Background:**

Plasma levels of amyloid‐β_1‐42/1‐40_, and glial fibrillary acidic protein (GFAP) have demonstrated predictive potential for amyloid pathology in the early stages of Alzheimer’s disease (AD) development. Utilizing baseline and up to 6‐year follow‐up plasma, positron emission tomography (PET) and cognitive data from cognitively unimpaired individuals, we here aim to test whether early changes in plasma biomarker levels associate with change in amyloid status and cognitive decline.

**Methods:**

From the EMIF‐AD PreclinAD study we selected individuals with normal cognition and longitudinal plasma, PET and cognitive data available (n_baseline_=200, table 1). Amyloid‐status was determined using visual assessment of dynamic [^18^F]flutemetamol‐PET scans. Plasma levels of amyloid‐β1‐40, amyloid‐β1‐42, neurofilament light (NfL), and GFAP were measured using the neurology 4‐plex E Simoa assay (Quanterix). Linear mixed models were applied to examine longitudinal changes in plasma biomarker levels, and their association with longitudinal changes in amyloid‐status and cognition.

**Results:**

At baseline 13.3%, and at 4‐year follow‐up 25.9% of participants were amyloid‐positive. Twenty‐one participants converted from a negative to positive amyloid‐status. At baseline, GFAP levels were higher and amyloid‐β_1‐42/1‐40_ lower in amyloid‐positive compared to negative individuals (β(SE)=0.43(0.14), β(SE)=‐0.75(0.21), p<0.005, respectively). In the total cohort, plasma levels of GFAP and NfL increased over time, whereas amyloid‐β_1‐42/1‐40_ remained stable (Figure 1). Although overall stable, plasma amyloid‐β_1‐42/1‐40_ decreased in participants that converted from a negative to positive amyloid‐status over time (β(SE)=‐0.04(0.02), p=0.02). Alongside, an increase in GFAP levels was observed in participants that converted from a negative to positive amyloid‐status compared to stable negative participants (Figure 1). No group differences were observed for NfL. A longitudinal increase in GFAP levels was associated with memory decline in stable amyloid positive individuals (GFAP*time*amyloid‐group status: β(SE)=‐0.09(0.02), p<0.001, Figure 2).

**Conclusions:**

Early changes in plasma GFAP and amyloid‐β_1‐42/1‐40_ are associated with changes in amyloid‐status in cognitively unimpaired individuals. Further, increases in plasma GFAP levels over time are predictive of future memory decline in amyloid‐positive individuals. These results support the potential of plasma GFAP and, to a lesser extent, amyloid‐β_1‐42/1‐40_ as diagnostic and prognostic markers for AD in early stages of disease development.